# Conductometric sensor for potassium ion profiling using lipophilic salt-incorporated non-toxic ion-selective membrane

**DOI:** 10.1557/s43578-025-01738-w

**Published:** 2025-11-18

**Authors:** Thiyagarajan Natarajan, Tom Wade, Anjana Ramesh Peringath, Diandian Zhang, Sohini Kar-Narayan

**Affiliations:** 1 Department of Materials Science, Device Materials Group, University of Cambridge, 27 Charles Babbage Road, Cambridge, CB3 0FS UK; 2 Materials Systems Engineering, Max Planck Institute for Dynamics of Complex Technical Systems, 39106 Magdeburg, Germany

**Keywords:** Membrane, Additive manufacturing, Sensor, Additives, Electrical properties

## Abstract

Conductometric ion-selective electrodes (ISEs) offer a promising alternative to conventional potentiometric ISEs due to their suitability for miniaturization, real-time monitoring, and reduced calibration requirements. Here, a non-toxic potassium-selective ionophore, 2-dodecyl-2-methyl-1,3-propanediyl bis[N-[5′-nitro(benzo-15-crown-5)-4′-yl]carbamate] (K-III/BME-44), was incorporated into a polyvinyl chloride (PVC)-based ion-selective membrane (ISM), along with the lipophilic salt potassium tetrakis(4-chlorophenyl)borate (KTpClPB). The membrane was deposited onto gold interdigitated electrodes to facilitate impedance-based measurements. Sensor performance was evaluated, demonstrating that the sensitivity, selectivity, and dynamic range of the ISE can be modulated by adjusting the concentration of KTpClPB. The optimized K-III-based ISEs exhibited a threefold higher response compared to the commonly used, but toxic, valinomycin-based counterpart under equivalent conditions. Selectivity coefficients, determined using both the Separate Solution Method (SSM) and the Fixed Interference Method (FIM), confirmed excellent potassium selectivity comparable to established potentiometric ISEs. These results highlight the potential of K-III-based conductometric ISEs for safe and accurate potassium detection in complex bioanalytical environments.

**Graphical abstract:**

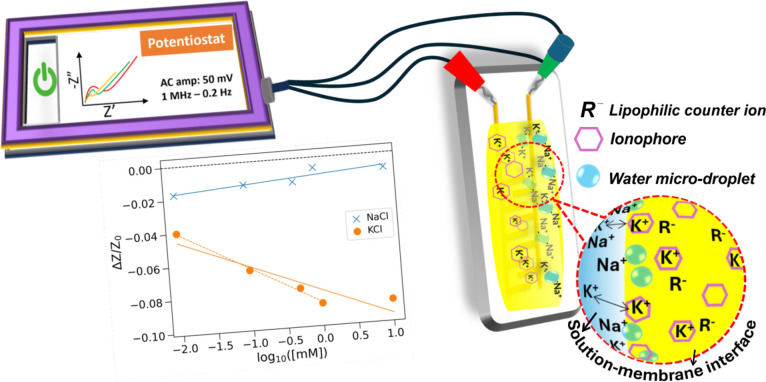

**Supplementary Information:**

The online version contains supplementary material available at 10.1557/s43578-025-01738-w.

## Introduction

Electrolytes play a vital role in maintaining physiological homeostasis and are essential for numerous bodily functions, such as hydration, nerve and muscle function, and cardiac rhythm regulation [1]. Electrolyte imbalance can lead to a range of health risks, from mild symptoms such as muscle cramps and fatigue to life-threatening conditions including cardiac arrhythmias and renal dysfunction. These imbalances are particularly prevalent in patients with chronic diseases such as kidney failure and cancer, where disruption of ion regulation is common [2–4]. A large population-based study in Rotterdam involving individuals aged ≥ 55 years reported that 15% of participants had at least one electrolyte disorder, with hyponatremia ([Na^+^] < 135 mmol/L) affecting 7.7% and hypernatremia ([Na^+^] > 145 mmol/L) affecting 3.4%, making these the most common abnormalities observed [5]. Beyond clinical relevance, quantification of electrolytes extends to areas such as agriculture, fishery, industrial control, food, water quality monitoring, and sport [6, 7].

Among electrolytes, potassium ions (K⁺) are particularly critical due to their role in cellular membrane potential, neuromuscular function, renal filtration, and cardiovascular regulation [8, 9]. Potassium imbalance is associated with dehydration [10], cardiovascular complications [11], and adverse effects from drugs such as Angiotensin-converting-enzyme inhibitors [12]. Despite its clinical relevance, no universal method exists for rapid or continuous potassium monitoring, especially outside of hospital environments [13, 14]. Similarly, the K^+^ concentration also plays a significant role in plant growth and metabolism. Both excess and deficiency of K^+^ were observed to affect nitrogen metabolism and carbon assimilation, while appropriate levels promote photo-assimilate transport, C and N metabolizing enzyme activities among others [15].

We recently proposed a purely electrical characterization of electrolytes in fluids based on impedance measurements [16, 17]. The impedance across frequencies at varying ion concentrations was measured, and the corresponding capacitance–frequency curves revealed a turning point frequency (TPF); an inflection in the capacitance–frequency curve might reflect the transition between low-frequency electrochemical double layer (EDL) capacitance and high-frequency geometric capacitance. Importantly, the TPF showed a strong linear correlation with ion concentration and this trend is highly reproducible with varying gradients for different cations, making it a reliable tool for fluid characterization [16]. However, its limitation lies in the lack of selectivity in a mixed ionic solution, which could be addressed by incorporating ion-selective membranes.

Traditional methods for potassium measurement utilize both spectroscopic and predominantly ion-selective electrodes (ISEs) in clinical diagnostics [14], soil nutrient analysis [18], and water quality control [19]. The conventional ISEs are highly accurate; however, they constitute an internal chamber filled with a solution containing a fixed concentration of the target ion which makes the miniaturization and long-term operational stability of the device challenging [6, 20].

In recent years, all-solid-state ISEs, also called solid-contact ISEs (SC ISEs), have emerged as promising alternatives, offering improved durability and potential for *in situ* or wearable integration [21–23]. These devices use solid contacts between the ion-selective membrane and a conductive substrate (carbon/metal-based electrodes) to enable ion-to-electron transduction [6]. Among transducer materials, conductive polymers such as polypyrrole, polyaniline, poly(3-octylthiophene) (POT), and Poly(3,4-ethylenedioxythiophene (PEDOT) are widely studied due to their redox activity and mixed conductivity, though their performance relies on stability, side reactions, and material properties such as morphology and dopant concentration [24, 25]. In contrast, nanostructured solid contacts generate interfacial potential via charge accumulation in the EDL, offering high capacitance, reduced potential drift, and improved stability through large surface area interfaces [6, 26]. However, unintended water accumulation at the membrane/solid-contact interface affects sensor performance [6, 25–27].

An emerging but less explored alternative is conductometry-based ISE, first assessed by Shulga et al. using a valinomycin-based K-ISE [28]. The advantage of conductometry-based sensors over SC-ISE is that they do not require a reference electrode, which simplifies the device construction and operation, and can be readily adopted for miniaturized and wearable platforms. This characteristic opens new avenues for continuous and on-the-go monitoring of electrolytes, particularly in non-clinical settings. Further, Day et al. evaluated the effect of valinomycin (ionophore) content on the sensor performance and observed 5 wt% ionophore to produce better sensor response compared to the commonly used 2 wt% ionophore content [29].

Despite its wider use in K-ISEs due to its selectivity, valinomycin poses toxicity concerns [30] which can be a hurdle in the deployment of wearable or implantable ISE for long-term continuous monitoring of K^+^ intended for better diagnosis and patient outcomes. To address this limitation, we have optimized the ISM composition with the non-toxic K^+^-ionophore; 2-Dodecyl-2-methyl-1,3-propanediyl bis[N-[5′-nitro(benzo-15-crown-5)-4′-yl]carbamate] (‘K-III’), for a conductometric-based K-ISE device.

Additionally, it was suggested that ISE membranes made with lipophilic cation exchanger, which provides electroneutrality and ensures permselectivity [31], does not exhibit a conductometric response [28]. Later, Mikhelson et al. showed that the ISE with lipophilic salt could simultaneously exhibit both potentiometric and conductometric response [32]. The conductometric response was proposed to originate from the water molecules adsorbed into the membrane, resulting in a rather less selective ionic response.

In this study, we have explored the effect of the lipophilic salt potassium tetrakis(4-chlorophenyl) borate in modulating the conductometric sensor performance. The sensor exhibited excellent responses to changes in potassium ion concentration even in the presence of twofold excess sodium ion concentration with selectivity on par with conductometric-based ISE prepared without lipophilic salt. The calculated selectivity (modified) coefficient values are comparable to the values reported for potentiometric ISEs. In addition, the experimental results demonstrated that the optimized sensor exhibited enhanced (~ 3x) sensitivity over a valinomycin-based ISE under similar test conditions, highlighting its potential for safe, accurate, and non-toxic K^+^ detection in biomedical and environmental applications.

## Results and discussion

### Optical and impedance characterization of ion-selective electrodes

**Figure 1:**
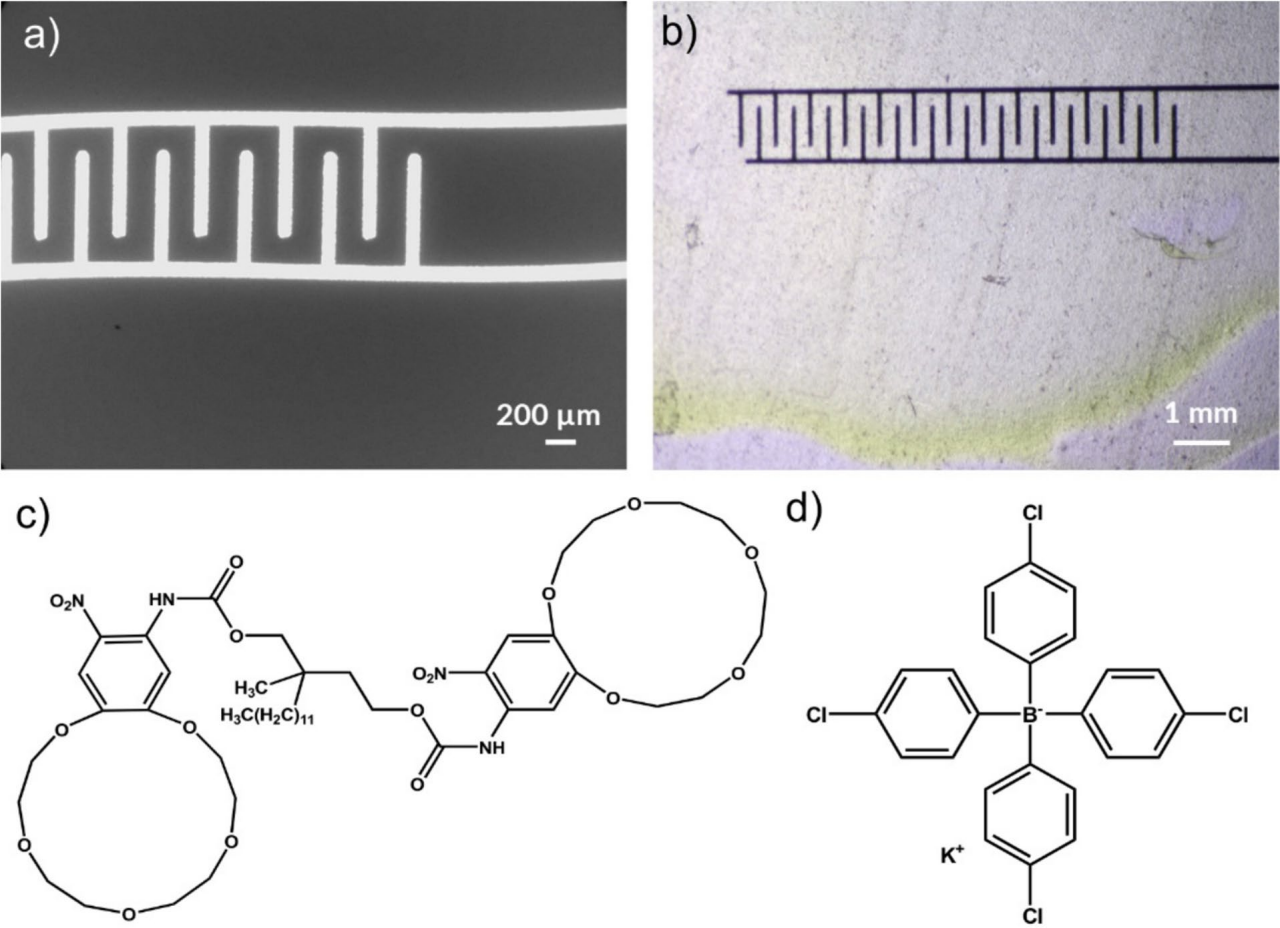
SEM image of the aerosol-jet printed interdigitated gold electrode (a). Optical microscopy image of ISM modified Au-IDEs (b). The chemical structures of K-III ionophore, 2-Dodecyl-2-methyl-1,3-propanediyl bis[N-[5′-nitro(benzo-15-crown-5)-4′-yl]carbamate], and lipophilic salt, KTpClPB, (c and d), respectively.

The aerosol-jet printed gold interdigitated (Au-IDEs) and ion-selective membrane-modified electrodes were characterized using scanning electron microscopy (SEM) and optical microscopy techniques. As shown in Fig. 1(a), the finger width of the interdigitated electrodes was roughly 100 µm, while the total width of the interdigitated electrode region was 1.2 mm. The distance between the centers of neighboring interdigitated fingers was ~ 100 μm. After drying of the drop-coated membrane, the modified electrode was subjected to optical image characterization. The image [Fig. 1(b)] shows the drop-coated ISM layer on top of the cured interdigitated gold electrodes. The thickness of the membrane was ~ 12 µm [Fig. S1, vide infra]. The chemical structures of K-III ionophore and KTpClPB are presented in Fig. 1(c and d).

Initially, the sensor without lipophilic salt was subjected to impedance measurement in 0.75 × PBS. As shown in the Nyquist plot (Fig. S2a), the signal was highly noisy, and the sensor exhibited a high impedance. We attribute this behavior to the high resistance of the membrane and probably to the lower conductivity of the printed electrode. The incorporation of lipophilic salts such as potassium tetrakis(4-chlorophenyl) borate (KTpClPB) is known to reduce membrane resistance by introducing co-diffusing ions.

However, the ISMs of conductometric sensors usually do not contain lipophilic salt, while it is typically added to potentiometric sensors. The primary reason is that the boundary potential consists of two components: the membrane potential and the interfacial potential. For the system to exhibit a Nernstian response, the ion exchange process at the membrane-solution interface should dominate, with the primary ion being exchanged, while the membrane composition remains constant. This is possible only when there is no coextraction of counterions into the membrane. This is achieved with the help of the lipophilic cation exchanger, and thus, a potentiometric sensor may not show a conductometric response and vice versa [32].

Shulga et al., who first reported the conductometric-based K-ISE without lipophilic salt, proposed coextraction as the reason for the observed linear change in conductivity with concentration [28]. Nevertheless, Mikhelson et al. demonstrated that a potentiometric sensor may simultaneously exhibit a conductometric response [32]. They proposed that the inclusion of water in the form of microdroplets within the membrane acted as a reservoir for extracted chloride ions, which served as sites for cation adsorption. This, in turn, led to a less selective and less sensitive ionic response and was identified as the primary reason for the observed change in conductivity.

**Figure 2:**
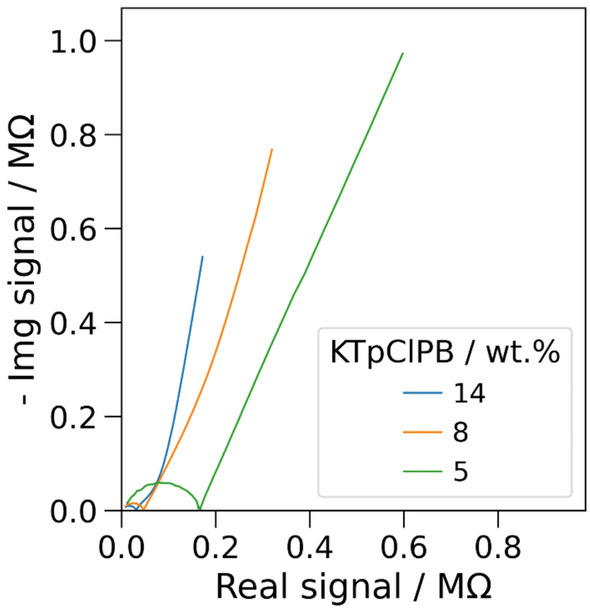
Nyquist plot of the impedance spectra obtained in the frequency range between 1 MHz and 200 mHz in 0.75 × PBS using K⁺-selective membranes (K-ISM) modified electrodes containing 5, 8, and 14 (wt%) KTpClPB, respectively.

Therefore, we investigated the effect of varying amounts of lipophilic salt KTpClPB in the membrane to determine whether this could improve the sensor response. With the addition of 1.1 wt% KTpClPB, a slight improvement in the sensor response was observed, especially at lower frequencies. However, the mid frequency region remained noisy, and the sensor response showed no clear trend with varying concentrations [Fig. S2(b-d)]. Encouraged by this improvement, we further increased the concentration of KTpClPB in the cocktail composition to ~ 5 wt%. As expected, the noise decreased substantially both in the higher and mid frequencies compared to 0 and 1.1 wt% KTpClPB. Further increase in the lipophilic salt content to ~ 8 and 14 wt% showed incremental improvement in the noise level (Fig. 2). Here, the semicircle at high frequency has been associated with the bulk resistance (*R*_m_) and geometric capacitance (*C*_geo_) of the membrane, and the response at mid- and low-frequency regions may reflect the charge transfer, related to ion exchange, and diffusion process, respectively.

**Figure 3:**
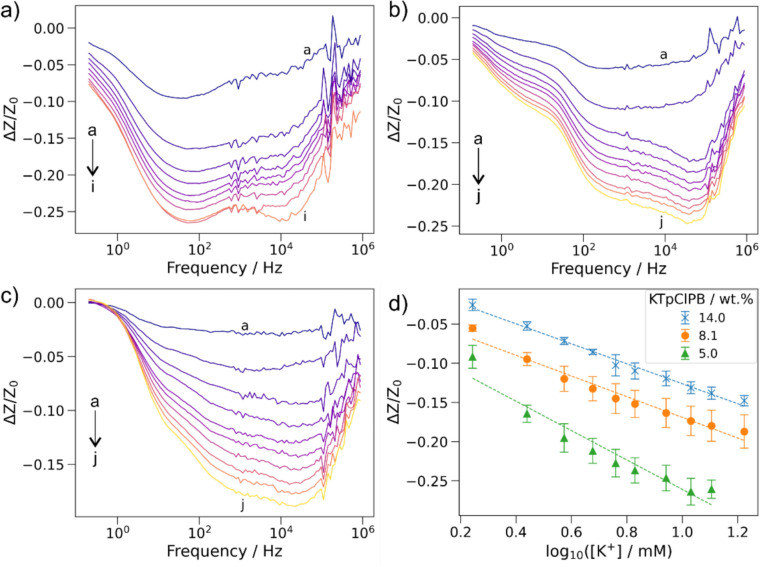
Influence of lipophilic KTpClPB salt on the sensor response: Plot of fractional difference (*ΔZ*/*Z*_0_) across the frequency range (200 mHz–1 MHz) with varying KCl concentration ((a-j): 1.75, 2.75, 3.75, 4.75, 5.75, 6.75, 8.75, 10.75, 12.75, 16.75 mM) (n = 3); (a) 5 wt% KTpClPB, (b) 8 wt% KTpClPB and (c) 14 wt% KTpClPB. (d) Calibration plot constructed by plotting the (*ΔZ*/*Z*_0_), measured at 100 Hz, vs. log [K^+^].

As the sensor device prepared with cocktails containing KTpClPB between 5 and 14 wt% showed significantly lower noise levels, we proceeded to measure the impedance response of these sensors. Upon systematic increase of KCl concentration, in the presence of fixed NaCl concentration (116.37 mM), the impedance (*Z*) decreased gradually. The calculated fractional difference ((Δ*Z*/*Z*_0_)/dec) values corresponding to various concentrations of potassium ions were plotted against the frequency range [Fig. 3(a–c)]. The calibration plot showed an excellent linear response for the change in concentrations with correlation coefficients (*R*^2^) ranging from 0.958 to 0.995, and the gradients (slope) between 0.13 and 0.19 ((*ΔZ*/*Z*_0_)/dec) at 100 Hz [Fig. 3(d)]. Overall, increasing lipophilic salt from 5 to 14 wt% in the membrane resulted in lower noise and wider dynamic range (1.75–10.75 mM and 1.75–16.75 mM for 5 wt% KTpClPB, 8 and 14 wt% KTpClPB, respectively) but the gradient (and therefore sensitivity) decreased with increasing lipophilic salt content. The slope of the sensor’s impedance response can be influenced by both interfacial charge transfer and bulk membrane resistance. At higher lipophilic salt content, the ionic strength of the membrane increases, enhancing the bulk conductivity and reducing the membrane resistance. The increased baseline conductivity may result in a smaller relative impact of the primary ion (K⁺) on total impedance. As a result, changes in ion activity led to smaller impedance variations, thereby reducing sensitivity. However, the system also exhibits a lower baseline noise and a smoother impedance response with respect to ion activity at higher KTpClPB concentrations. This stability allows even small changes in ion concentration to be detected, effectively broadening the dynamic range despite the reduced gradient. The potassium ion concentration in sweat is present in range of 3–15 mM [33], which falls well within the demonstrated range of our sensor (1.75–16.75 mM) and thus confirms the applicability of the sensor for sweat monitoring.

### Sensor selectivity

**Figure 4:**
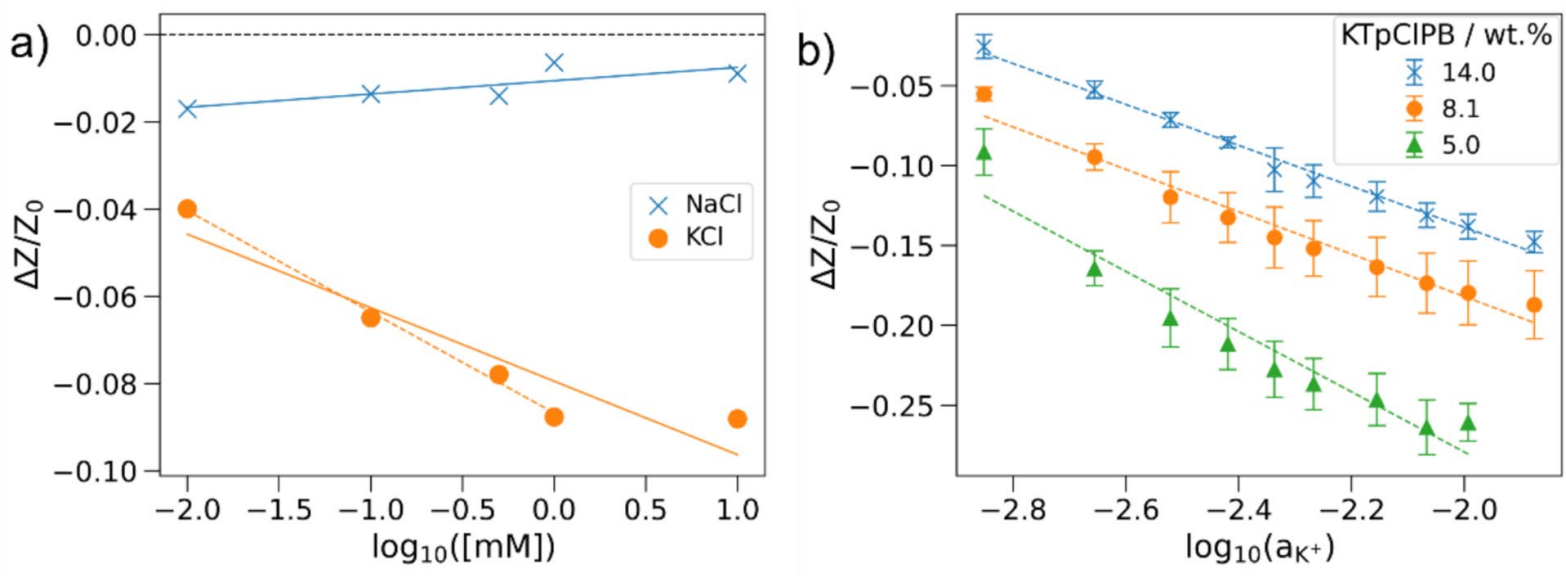
(a) Plot of fractional response vs. log [K^+^] obtained from the separate solution method: The impedance response of varying concentrations of KCl and NaCl (0.01–10 mM) were measured separately. (b) Plot of fractional response vs. log [a_K_^+^], measured at 100 Hz, obtained from the fixed interference method: [NaCl] = 116 mM, KCl concentration varied between 1.75 and 16.75 mM.

The selectivity of the sensor is paramount for the reliable and accurate measurement of electrolytes, especially in competitive or mixed-ion environments such as biological fluids. High selectivity ensures that the sensor can distinguish the target ion, in this case potassium (K⁺), from potentially interfering ions such as sodium (Na⁺), which may be present in much higher concentrations in physiological samples. To evaluate the selectivity of the ISEs, we employed both the Separate Solution Method (SSM) and the Fixed Interference Method (FIM), following IUPAC guidelines [34]. In conventional SSM potentiometric measurements, the electromotive force (EMF) of two separate solutions, one containing the primary ion (A, K^+^) and the other the interfering ion (B, Na^+^), are measured individually. The potential is plotted against the logarithm of activity for each ion, and the potentiometric selectivity coefficient K_A,B_ is determined from the activities that correspond to the same EMF value. We adapted a slightly modified approach for impedance-based measurements, using a membrane containing 8 wt% KTpClPB. Instead of EMF, we plotted the fractional impedance difference against the logarithmic concentration of K⁺ and Na⁺ [Fig. 4(a)]. The slopes obtained were −0.023 for K⁺ and 0.003 for Na⁺, indicating a much higher response to potassium compared to sodium. It is worth noting that in traditional potentiometric ISEs, the concentration of lipophilic salt is typically kept below 70 wt% of the ionophore concentration to avoid saturation effects and minimize non-specific ion exchange. Excessive salt content can compromise selectivity by promoting random ion transport, thus acting as a non-specific ion exchanger and distorting the sensor output [35]. Despite these considerations, our impedance-based sensor showed only a minor response to NaCl, demonstrating that selectivity was preserved. In fact, the Na⁺ response was ~ 13% of the K⁺ response, closely matching the value of 8.3% for ISE fabricated without lipophilic salt [29].

To further assess selectivity under practical conditions, such as in sweat, where Na⁺ is present at much higher concentrations, the FIM approach was also applied. Here, the Na⁺ concentration was fixed at 116 mM, while the K⁺ concentration was varied [Fig. 4(b)]. Fractional impedance differences at 100 Hz were plotted against the logarithm of K⁺ activity, and the intercept point was used to extract a_A_, as per IUPAC recommendations [34]. The selectivity coefficient was calculated using the modified equation:

1 $$\log K_{{K^{ + } ,Na^{ + } }}^{{{\mathrm{Im}} p}} = \frac{{a_{A} }}{{(a_{B} )^{{{\raise0.7ex\hbox{${Z_{A} }$} \!\mathord{\left/ {\vphantom {{Z_{A} } {Z_{B} }}}\right.\kern-0pt} \!\lower0.7ex\hbox{${Z_{B} }$}}}} }},$$

where a_A_ and a_B_ are the activities and Z_A_ and Z_B_ are the charges of the primary and interfering ions, respectively.

The calculated log K_K,Na_ values were − 2.5, − 2.4, and − 2.1 for membranes containing 5, 8, and 14 (wt%) KTpClPB, respectively, comparable to values reported in literature [13, 36, 37]. Although water droplets in the membrane could trap counterions and form microdomains that facilitate cation hopping [32], given the heterogeneous nature of these microdomains in the membrane, the presence of ionophores more likely promotes preferential diffusion of the primary ion, thereby retaining selectivity. While excess lipophilic salt may act as a poorly selective cation exchanger, the coordinated transport via the ionophore still appears to dominate.

### Comparison of K-III and K-I (Valinomycin) performance

**Figure 5:**
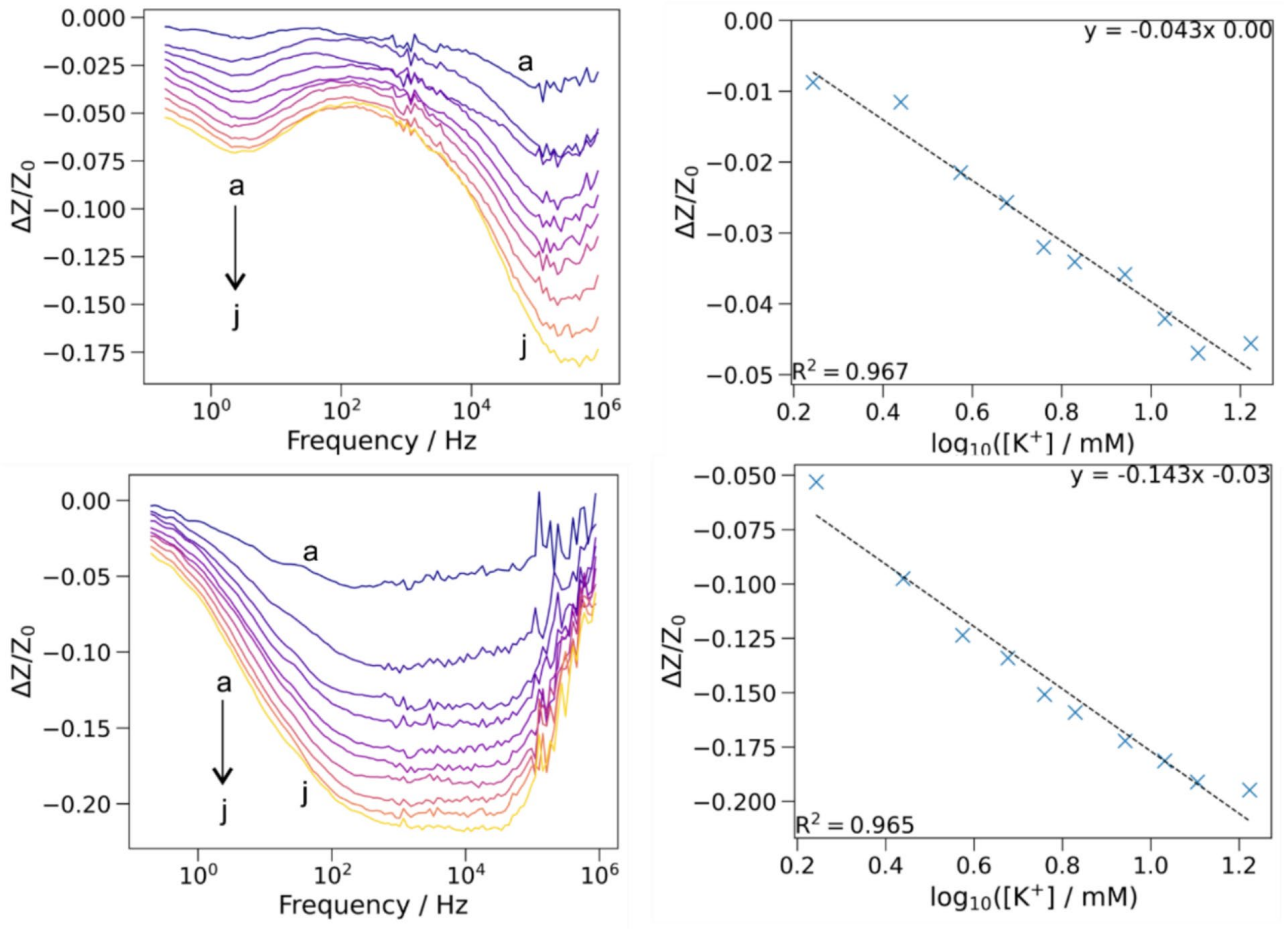
(Left-top and bottom) Plot of fractional difference response (*ΔZ*/*Z*_0_) across the frequency range with varying KCl concentration ((a-j): 1.75, 2.75, 3.75, 4.75, 5.75, 6.75, 8.75, 10.75, 12.75, 16.75 mM) corresponding to K-I and K-III ionophore modified ISEs, respectively. (Right-top and bottom) The corresponding calibration plot; (*ΔZ*/*Z*_0_) vs. log [K^+^], measured at 100 Hz

Since K-I ionophore is predominantly used in K-ISEs, we prepared a K-I ionophore containing ISE with 9 wt% KTpClPB content. Similar to the K-III ionophore-based measurement, 116 mM NaCl was used as background electrolyte. Upon changing the KCl concentration, the sensor showed a continuous decrease in the measured impedance response. Further, the fractional difference of impedance plotted against logarithmic concentration of KCl showed a linear decrease in impedance (*R*^2^ = 0.967) and yielded a gradient of −0.043 (*ΔZ*/*Z*_0_)/dec [Fig. 5(a and b)]. However, the ISE containing the K-III ionophore showed a linear response (*R*^2^ = 0.965) with a gradient of −0.143 (*ΔZ*/*Z*_0_)/dec [Fig. 5(c and d)], which is ~ 3 times higher than that of the ISE with K-I ionophore. This distinct enhancement in gradient demonstrates the superior K^+^ response of K-III-based ISM. Given the larger Na^+^ concentration and complexity of sweat, a sensor with higher sensitivity for K^+^ would enable superior sensing. The improved performance of K-III ionophore modified sensor over K-I could partly be attributed to the structural geometry of K-III. The K-III ionophore has a long alkyl chain, similar to surfactants, that has been suggested to localize more favorably near the membrane/solution interface (i.e., “active zone” where K⁺ exchange occurs), amplifying the interfacial exchange process [38].

## Conclusion

This study establishes that impedance-based ion-selective electrodes (ISEs) incorporating K-III ionophore with varying concentrations of the lipophilic salt potassium tetrakis(4-chlorophenyl) borate (KTpClPB) exhibit tunable analytical performance in terms of sensitivity and dynamic range. Notably, ion selectivity toward K⁺ over Na⁺ was maintained, as confirmed by both the Separate Solution and Fixed Interference Methods. The sensor’s ability to detect potassium selectively in mixed electrolyte solutions and a threefold higher response than the conventional valinomycin (K-I) is particularly advantageous in detecting K⁺ in sweat, where high Na⁺ levels and complex ionic backgrounds often obscure accurate potassium quantification. Overall, the results underscore the viability of impedance-mode ISE incorporating non-toxic ionophores for sensitive, selective, and safe K⁺ detection, unlike Valinomycin, in complex biological matrices. These findings can assist in the future development and integration of such sensors into bioanalytical platforms aimed at real-time electrolyte monitoring in clinical and wearable technologies, thus enhancing the reliability of personalized health monitoring systems, minimizing ionophore toxicity, and enabling long-term non-invasive monitoring of electrolytes in dynamic physiological conditions. However, it should be noted that KTpClPB is not classified as non-toxic; exposure may cause irritation to the skin, eyes, and respiratory tract, and its effects on living cells have not been comprehensively evaluated. Therefore, direct contact between KTpClPB-containing materials and biological tissues should be approached with caution. For potential biomedical use, non-toxic lipophilic salts or encapsulation strategies with established biocompatibility, such as polyurethane, that has shown lower leaching of ISM components could be considered to mitigate safety risks arising from leaching of the ISM components[39].

## Materials and methods

### Materials

Bis(2-ethylhexyl)sebacate (DOS), valinomycin (K-I), 2-Dodecyl-2-methyl-1,3-propanediyl bis[*N*-[5′-nitro(benzo-15-crown-5)-4′-yl]-carbamate] (K-III/BME-44), potassium tetrakis(4-chlorophenyl) borate (KTpClPB) are of Selectophore grade and obtained from Sigma Aldrich. Tetrahydrofuran (THF, > 99.5 wt%) was purchased from Fisher. High molecular weight Poly(vinyl chloride) (PVC) and microscope slides-clear ground (0.8–1.0 mm) were purchased from Fisher. Gold ink-UTDAu25 TE gold nanoink (25 wt% w/v solution in proprietary organic solvents) was obtained from UT Dots, Inc., Illinois, USA. Conductive silver epoxy (8331-14G) was obtained from MG Chemicals, Canada. Deionized water (Purite, UK, (σ < 10 μS)) was used for preparing the aqueous stock solutions. Stock solutions of KCl, NaCl, and CaCl_2_, obtained from Sigma Aldrich, were prepared in the deionized water. 1 × phosphate buffer saline (PBS), containing 155.17 mM NaCl, 2.96 mM Na_2_HPO_4_. 7H_2_O, and 1.05 mM KH_2_PO_4_, was purchased from Gibco™ PBS, pH 7.4, Fisher Scientific (Catalog number 10010031).

### Ion-selective membrane cocktail

The K^+^-ion-selective membrane cocktail was prepared in THF solvent. The required amounts of neutral ionophores K-III or K-I (~ 2–5 wt%) and the lipophilic salt KTpClPB (~ 0–14 wt%) were taken in a glass vial. The plasticizer DOS (66 wt%) was added to the vial, and the mixture was dissolved in 1 mL of THF using a vortex mixer. Finally, high molecular weight PVC (33 wt%) was added to the above mixture and vortexed again to obtain a clear solution. This mixture was left stirring overnight to ensure complete dissolution and mixing of all the components.

### Device fabrication

#### Aerosol-jet printing of gold interdigitated electrodes

**Figure 6:**
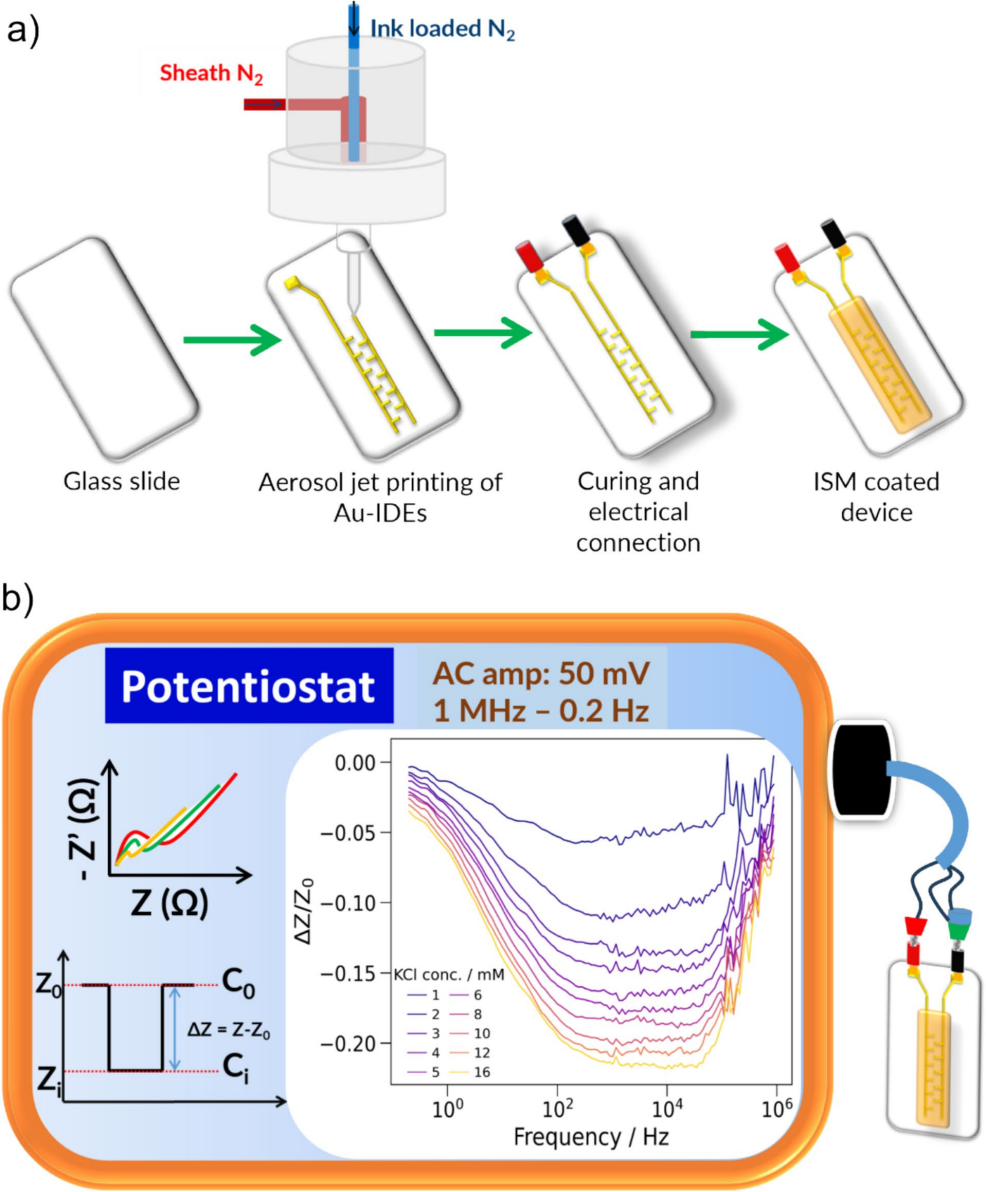
(a) Device fabrication process: aerosol-jet printing of gold interdigitated electrodes followed by electrode surface modification for integration of the ion-selective membrane (ISM) layer. (b) Impedance measurement setup and corresponding sensor response.

The glass slides were cleaned with isopropanol. The aerosol-jet printer (Optomec AJ200, Optomec Inc., New Mexico, USA), equipped with a UA Max ultrasonic atomizer to atomize the ink, was employed for electrode printing. Gold interdigitated electrodes (Au-IDE), using UTDAu25 TE gold nanoink, containing 25 wt% gold nanoparticles with an average particle size of 3–5 nm, and a viscosity of ~ 3 cP, were aerosol-jet printed on the cleaned glass slide (Fig. 6). The electrode patterns were made using AutoCAD. A 300 μm diameter tip was used with a working distance of ~ 2 mm. The ultrasonic atomizer operated at a current between 0.5 and 0.6 A, with the cooling water temperature maintained at 20 °C. The substrate platen temperature was set to 90 °C. The printing speed was 3 mm/s. The ink and sheath flow rates were typically approximately 30 and 120 sccm, respectively, and were manually set and adjusted throughout printing to maintain print quality due to printer drift over time. The interdigitated region of the electrodes was either 5 mm or 20 mm long. After curing (2 h at 200 °C), conductive silver epoxy was applied to attach wires to the printed contact pads. After curing of the epoxy (65 min at room temperature), the ISM cocktail was drop-coated on to the working area of the Au-IDEs [Fig. 6(a)]. The electrode was left to dry overnight to ensure complete drying. The thickness of the ISM membrane was measured using a Dektak XT profilometer (Bruker, Massachusetts, USA). The average height of the printed Au- interdigitated electrodes was measured using the profilometer prior to ISM deposition, and the value was found to be 0.65 µm. Similarly, the thickness of the drop-coated ion-selective membrane was also measured, which provided an average thickness of 12 µm (Fig. S1). The impedance measurement setup and sensor response of the device are illustrated in Fig. 6(b).

### Impedance measurement

Impedance measurements were performed using the BiPotentiostat (Palmsens4, maximum impedance 5 GΩ) with a two-electrode setup. The frequency scan was performed between 1 MHz and 200 mHz, and an AC excitation amplitude of 50 mV was used, with zero DC offset. A 0.75 × PBS solution was used as a background electrolyte. No presoaking was performed in either NaCl or KCl solution prior to measurements. The impedance response in the presence of background electrolyte stabilized after a couple of measurements (~ 15 min). The sensor response was assessed using the fractional difference method, as shown in Fig. 1(b), where $${\raise0.7ex\hbox{${\Delta Z}$} \!\mathord{\left/ {\vphantom {{\Delta Z} {Z_{0} }}}\right.\kern-0pt} \!\lower0.7ex\hbox{${Z_{0} }$}}$$ is plotted against logarithmic concentration of K^+^. The fractional difference method directly captures analyte-induced changes independent of baseline variability, making it valuable for sensors affected by drift or aging and especially for identifying optimal response frequencies across the spectrum.

## Supplementary Information

Below is the link to the electronic supplementary material.

Supplementary file1 (PDF 359 KB)

## Data Availability

The data that supports the findings of this study are available at the University of Cambridge Apollo data repository (DOI: 10.17863/CAM.123052).
