# Supplementary material for: Conductometric sensor for potassium ion profiling using lipophilic salt-incorporated non-toxic ion-selective membrane
Source: J Mater Res. 2025 Nov 18;40(23):3297–307. doi: 10.1557/s43578-025-01738-w (PMC12717214; doi:10.1557/s43578-025-01738-w)
Supplement: Supplementary file 1 — Supplementary file1 (PDF 359 KB) [file 43578_2025_1738_MOESM1_ESM.pdf]

Supplementary information

Conductometric Sensor for Potassium Ion Profiling Using Lipophilic Salt-Incorporated Non-Toxic Ion-Selective Membrane

Thiyagarajan Natarajan[1,*], Tom Wade[1], Anjana Ramesh Peringath[1], Diandian Zhang[1], Sohini Kar-Narayan[1,2*]

[1]Department of Materials Science, Device Materials Group, University of Cambridge, Cambridge, CB3 0FS, United Kingdom

[2]Materials Systems Engineering, Max Planck Institute for Dynamics of Complex Technical Systems, 39106 Magdeburg, Germany

Conductivity measurement

The resistance was measure using the Sciospec ISX3v2 Impedance Analyser (Sciospec Scientific Instruments GmbH, Germany). Electrical conductivity (σ) was determined using the relationship $\sigma = {}^{L}/_{RA}$, where R is the measured resistance, A is the cross-sectional area (obtained via profilometry), and L represents the fixed length of the measured region, which is 5 mm. The conductivity of the Au-IDE was calculated to be 51510 S/m.

Figure S1

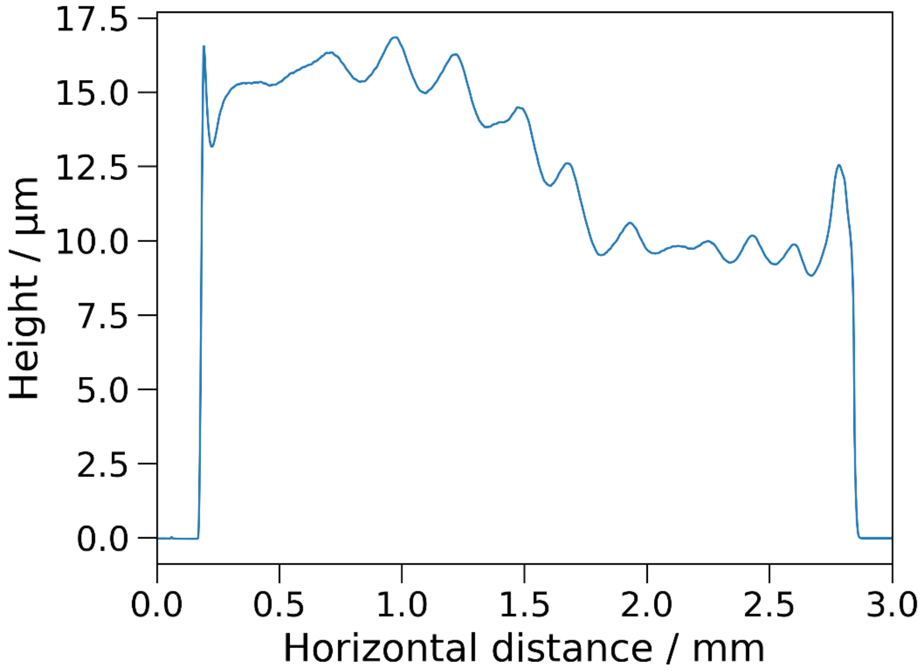

Figure S1 The measurement of the ISM membrane height profile performed with Dektak XT profilometer. The average thickness was found to be ~12 µm.

Figure S2

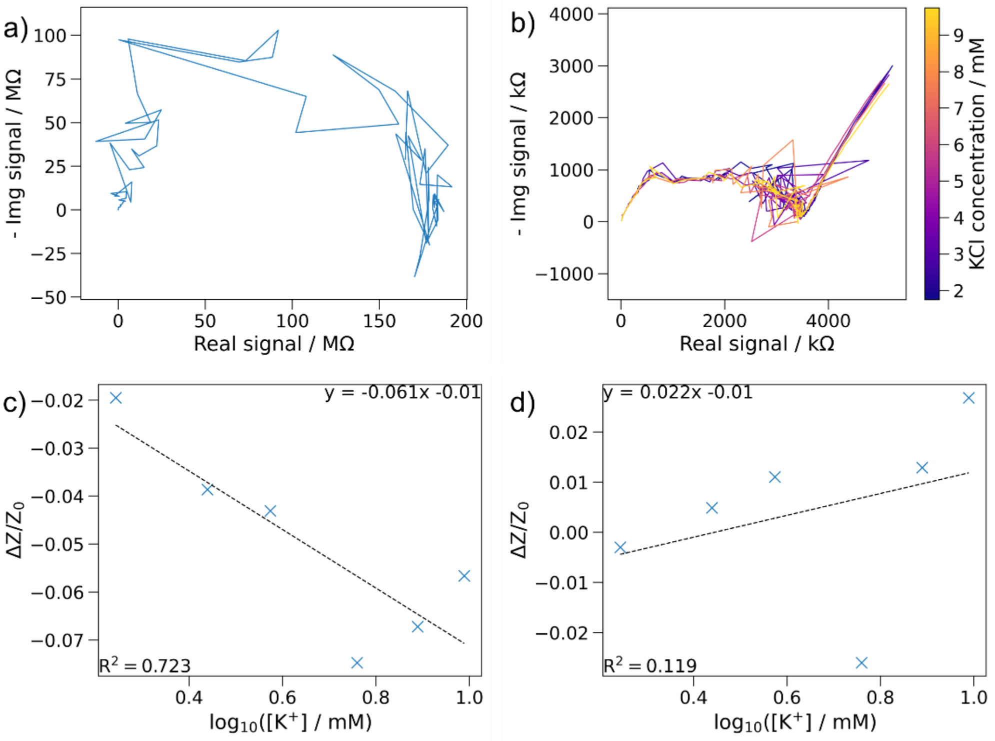

Figure S2 (a) Nyquist plot of the impedance spectra recorded in 0.75 x PBS for the ion-selective electrode (ISE) without lipophilic salt (0 wt% KTpClPB). (b) Nyquist plot for the ISE containing 1.1 wt% KTpClPB under identical conditions, measured across increasing KCl concentrations (1.75–8.75 mM). (c, d) Fractional difference response ($\Delta Z/Z_0$) plotted against the logarithm of potassium ion concentration $[K^+]$ (1.75–8.75 mM), measured at 100 Hz (c) and 0.2 Hz (d), respectively.
